# Supplementary material for: Spatial Variation in Stream-Water Bacterial Communities Across Sites Differing in Wild Chinese Giant Salamander Detection Frequency
Source: Biology (Basel). 2026 Sep 18;15(18):1647. doi: 10.3390/biology15181647 (PMC13604818; doi:10.3390/biology15181647)
Supplement: Supplementary file 1 [file biology-15-01647-s001.zip › Data Related to “Spatial Variation in Stream-Water Bacterial Communities across Sites Differing in Wild Chinese Giant Salamander Detection Frequency”/Microbial Data/result/08.FunctionPrediction/picrust2/heatmap/sample/heatmap.sample.pathways.cluster.pdf]

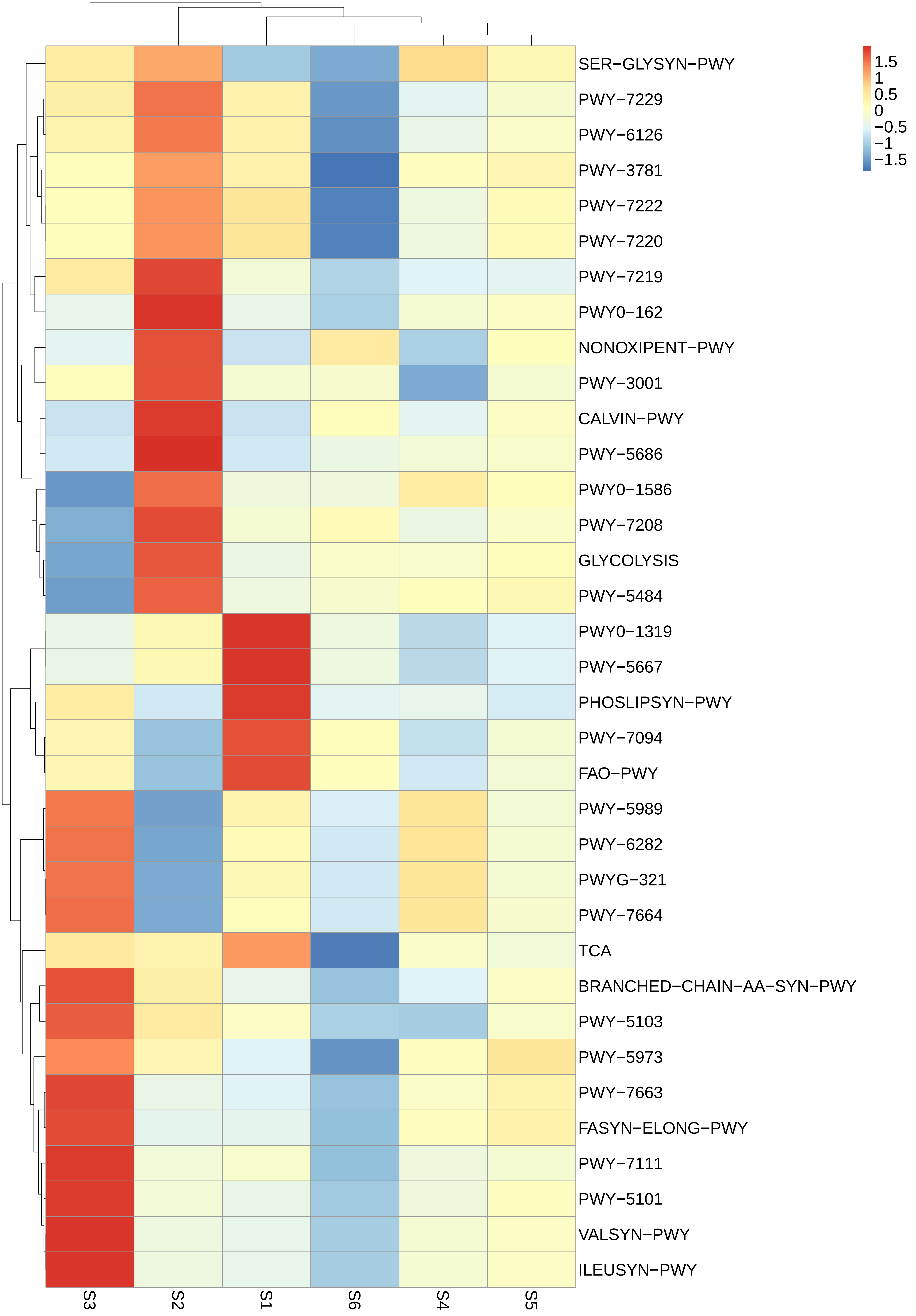

1.5
1
0.5
0
−0.5
−1
−1.5
SER−GLYSYN−PWY
PWY−7229
PWY−6126
PWY−3781
PWY−7222
PWY−7220
PWY−7219
PWY0−162
NONOXIPENT−PWY
PWY−3001
CALVIN−PWY
PWY−5686
PWY0−1586
PWY−7208
GLYCOLYSIS
PWY−5484
PWY0−1319
PWY−5667
PHOSLIPSYN−PWY
PWY−7094
FAO−PWY
PWY−5989
PWY−6282
PWYG−321
PWY−7664
TCA
BRANCHED−CHAIN−AA−SYN−PWY
PWY−5103
PWY−5973
PWY−7663
FASYN−ELONG−PWY
PWY−7111
PWY−5101
VALSYN−PWY
ILEUSYN−PWY
S3
S2
S1
S6
S4
S5
